# Effect of exercising at minimum recommendations of the multiple sclerosis exercise guideline combined with structured education or attention control education – secondary results of the step it up randomised controlled trial

**DOI:** 10.1186/s12883-017-0898-y

**Published:** 2017-06-24

**Authors:** Susan Coote, Marcin Uszynski, Matthew P. Herring, Sara Hayes, Carl Scarrott, John Newell, Stephen Gallagher, Aidan Larkin, Robert W Motl

**Affiliations:** 1 Department of Clinical Therapies, University of Limerick, Limerick, Ireland; 2 Health Research Institute, University of Limerick, Limerick, Ireland; 3 Multiple Sclerosis Society of Ireland, Western office, Galway, Ireland; 4 Department of Physical Education and Sports Science, University of Limerick, Limerick, Ireland; 5 HRB Clinical Research Facility, National University of Ireland, Galway, Ireland; 6 School of Mathematics and Statistics, University of Canterbury, Christchurch, New Zealand; 7 School of Mathematics, Statistics and Applied Mathematics, National University of Ireland, Galway, Ireland; 8 Department of Psychology, University of Limerick, Limerick, Ireland; 9 Department of Physical Therapy, School of Health Professions, University of Alabama at Birmingham, Birmingham, USA

**Keywords:** Multiple sclerosis, Exercise, Fatigue, Cognition, Behaviour change techniques, Social cognitive theory, Randomised controlled trial

## Abstract

**Background:**

Recent exercise guidelines for people with multiple sclerosis (MS) recommend a minimum of 30 min moderate intensity aerobic exercise and resistance exercise twice per week. This trial compared the secondary outcomes of a combined 10-week guideline based intervention and a Social Cognitive Theory (SCT) education programme with the same exercise intervention involving an attention control education.

**Methods:**

Physically inactive people with MS, scoring 0–3 on Patient Determined Disease Steps Scale, with no MS relapse or change in MS medication, were randomised to 10-week exercise plus SCT education or exercise plus attention control education conditions. Outcomes included fatigue, depression, anxiety, strength, physical activity, SCT constructs and impact of MS and were measured by a blinded assessor pre and post-intervention and 3 and 6 month follow up.

**Results:**

One hundred and seventy-four expressed interest, 92 were eligible and 65 enrolled. Using linear mixed effects models, the differences between groups on all secondary measures post-intervention and at follow-up were not significant. Post-hoc, exploratory, within group analysis identified improvements in both groups post intervention in fatigue (mean ∆(95% CI) SCT -4.99(−9.87, −0.21), *p* = 0.04, Control −7.68(−12.13, −3.23), *p* = 0.00), strength (SCT -1.51(−2.41, −0.60), *p* < 0.01, Control −1.55(−2.30, −0.79), *p* < 0.01), physical activity (SCT 9.85(5.45, 14.23), *p* < 0.01, Control 12.92(4.69, 20.89), goal setting (SCT 7.30(4.19, 10.4), *p* < 0.01, Control 5.96(2.92, 9.01), *p* < 0.01) and exercise planning (SCT 5.88(3.37, 8.39), *p* < 0.01, Control 3.76(1.27, 6.25), *p* < 0.01) that were maintained above baseline at 3 and 6 month follow up (all *p* < 0.05). Only the SCT group improved at 3 and 6 month follow up in physical impact of MS(−4.45(−8.68, −0.22), −4.12(−8.25, 0.01), anxiety(−1.76(−3.20, −0.31), −1.99(−3.28, −0.71), depression(−1.51(−2.89, −0.13), −1.02(−2.05, 0.01)) and cognition(5.04(2.51, 7.57), 3.05(0.81, 5.28), with a medium effect for cognition and fitness (Hedges’ g 0.75(0.24, 1.25), 0.51(0.01, 1.00) at 3 month follow up.

**Conclusions:**

There were no statistically significant differences between groups for the secondary outcomes once age, gender, time since diagnosis and type of MS were accounted for. However, within the SCT group only there were improvements in anxiety, depression, cognition and physical impact of MS. Exercising at the minimum guideline amount has a positive effect on fatigue, strength and PA that is sustained at 3 and 6 months following the cessation of the program.

**Trial registration:**

ClinicalTrials.gov, NCT02301442, retrospectively registered on November 13th 2014.

## Background

Multiple sclerosis (MS) is a chronic and often progressive condition affecting the central nervous system. MS has many consequences, including impaired strength, fitness, mood, fatigue and cognition, along with limitations of activities such as walking that impact on quality of life. Available evidence supports the beneficial effects of exercise on fatigue [1, 2], depression [3] fitness [4], walking mobility [5, 6], in addition to quality of life [7]. Indeed, this evidence has led to the development of the MS Exercise guideline [8, 9] which recommends moderate intensity aerobic exercise for 30 min and resistance training involving major muscle groups twice weekly.

We are not aware of a single trial that has actually documented the benefits of the exercise guidelines in MS. Of further concern, there are few studies in the MS exercise literature that have evaluated the long-term benefits of exercise interventions, and the results are mixed. For example, we reported positive improvements from a combined aerobic and resistance exercise programme in the community [10]; however, the improvements generally were not maintained 12 weeks post-intervention [11], suggesting that additional measures are required to enable sustained increases in physical activity behaviour among PwMS. This need to foster long-term exercise participation is not unique to PwMS and authors have highlighted the need to include theory-based behaviour change interventions [12]. Social cognitive theory has been extensively investigated among PwMS, and exercise self-efficacy and goal setting are consistently associated with [13] and predictive of [14] physical activity behaviour. Indeed a recent meta-analysis demonstrated significant associations of these constructs and outcome expectancies with physical activity [15].

We have conducted a series of clinical trials (i.e., Phase I and II) with relatively small samples for examining the efficacy of an Internet-delivered behavioural intervention based on social cognitive theory (SCT) for increasing physical activity among ambulatory persons with MS [16–19]. Our most recent trial included the website and one-one-one video coaching and demonstrated moderate to large improvements in minutes/day of moderate/vigorous physical activity, endurance walking performance, information processing speed, symptoms of fatigue, depression, anxiety, and pain, and quality of life (QOL) over a six-month period [20]. Collectively, such data support the efficacy of the behavioural intervention for increasing and sustaining physical activity in PwMS and possibly improving walking, cognition**,** symptoms, and QOL outcomes.

The Step it Up study [21] combined the collective knowledge and expertise gained from the Irish community exercise programme with the U.S. online intervention. The 10-week programme firstly aimed to enable inactive PwMS to reach the recently published aerobic and resistance exercise guidelines. We further investigated how embedding this exercise programme in a structured SCT-based education intervention compared to an attention-control education intervention. The current paper reports the results for the secondary outcomes of MS symptoms, physical activity, and SCT constructs. The primary outcome and feasibility metrics, presented elsewhere (Hayes et al. in press) demonstrated that both groups improved significantly in the primary outcome, the six minute walk test (6MWT), and this improvement was maintained at 6 month follow-up. An exploratory analysis of those with three of four assessments demonstrated that the SCT group had a ~ 40 m greater improvement in 6MWT than the control group post-intervention and at 6-month follow up (*p* = 0.04 for both).

## Methods

### Design

This was a multicentre, double blind, randomised controlled trial (RCT).

### Setting and participants

Participants were recruited through the MS Society of Ireland, and via neurology clinics in three urban locations in the Republic of Ireland. Details of the recruitment process are further detailed in the protocol paper [21]. Inclusion criteria were: (1) physician-confirmed formal diagnosis of MS, (2) aged 18 years or more, (3) Patient Determined Disease Steps (PDDS) scale score of 0–3, (4) a sedentary lifestyle (<30 min of moderate to strenuous exercise one day or more per week over the last six months) and (5) willing to give written informed consent. Exclusion criteria were: (1) pregnancy, (2) MS relapse in the previous 12 weeks and (3) changes to MS medication or steroid treatment in the previous 12 weeks. Participants were sent the consent form in advance of the baseline assessment, and written consent was obtained in person by a blinded assessor.

### Randomisation and blinding

Participants were randomly allocated into the exercise plus SCT-based intervention or the exercise plus attention control education intervention. Random allocation procedures have been previously outlined [21] and were adhered to. JN generated the random allocation sequence, SH enrolled participants, and SC assigned participants to interventions. The outcome assessor (SH) was blind to allocation throughout the study as were the statisticians (CS, JN). All participants were informed that the study aimed to examine the effect of combining exercise and education, and therefore were blinded regarding group allocation.

### Screening questionnaire

Potential participants were screened for eligibility for this study using a questionnaire that included the Patient Determined Disease Steps (PDDS) scale [22], confirmation from participant of MS diagnosis and questions regarding PA levels that have been detailed elsewhere [21].

### Outcomes

Outcome measures were conducted pre-intervention post-intervention and at 3 and 6 month follow-up.

At baseline, participants provided demographic details and a researcher formally trained in the use of the Expanded Disability Status Scale (EDSS [23]) (SH) administered the EDSS to all participants at baseline. MS diagnosis according to the McDonald or Poser criteria was confirmed in writing from the participant’s consultant neurologist.

The SenseWear Arm band (SWA) provided an objective estimate of PA [24] using both mean daily step count and mean daily energy expenditure estimates over a 7-day period. The 5 times sit to stand test (5xSTS) [25], the Modified Canadian Aerobic Fitness Test (mCAFT) [26] and the Godin Health Index of the Godin Leisure-Time Exercise Questionnaire (GLTEQ) [27] measured lower extremity muscle strength, aerobic capacity and PA behaviour, respectively. The Hospital Anxiety and Depression Scale (HADS) [28], Symbol Digit Modalities Test (SDMT) [29], Multiple Sclerosis Impact Scale 29 (MSIS-29) [30], and Modified Fatigue Impact Scale (MFIS) [31] measured depression, anxiety, cognitive processing speed, impact of MS and fatigue, respectively. Five questionnaires were implemented to measure SCT domains. These included the Exercise Self-Efficacy Scale (EXSE) [32], Exercise Goal Setting (EGS) scale [33], Multidimensional Outcomes Expectations for Exercise Scale (MOEES) [34], Social Provisions Scale (SPS) [35], and Exercise Benefits and Barriers questionnaire [36]. These measures and associated psychometric properties have been described in the trial protocol [21].

### Interventions

The exercise intervention was common to both groups and was delivered by physiotherapists. The aim of the exercise component was to progressively increase the intensity of both aerobic and strengthening exercise to enable the participants to reach the published exercise guidelines for people with mild-to-moderate MS [37], and has been previously described in detail [21]. Over the 10-week programme participants attended the group exercise class on six occasions, supplemented with a telephone coaching call in the weeks without classes (intervention weeks 4, 6, 7 and 9). After each of the group exercise classes the attention control group received an education session about topics unrelated to PA behaviour, e.g. diet, vitamin D, sleep, temperature and hydration, and immunisations and vaccinations. The exercise plus SCT-based intervention group received a similar duration of education based on the principles of SCT for health behaviour change, namely: self-efficacy, outcome expectations, goal-setting, barriers and benefits and has been previously described [21].

### Analysis

The study was powered for the primary outcome, 6MWT and consistent with data from a large international study [38], it was assumed that the effect of the intervention would yield an average improvement in 6MWT distance of 36 m with an estimated standard deviation of 48.2 m. In order to have 80% power (at the 5% significance level) to detect such a difference in mean improvement in 6MWT over the study period between groups, a sample of size 62 randomised equally to two arms (i.e. 31 per arm) was needed.

Suitable numerical statistics and graphical summaries were used to describe characteristics of the sample at baseline and to assess the validity of any distributional assumptions needed for the formal analysis. All tests of significance were two-sided and conducted at an alpha = 0.05 level of statistical significance.

The statistical modelling compared differences in the longitudinal response variables between the two intervention arms at each of the three post-intervention follow-ups while correcting for the baseline measurements for each participant. A linear mixed model for a continuous response over time due to the two interventions, whilst adjusting for participant-specific covariates and factors; namely the response of interest at baseline, age, gender, time since diagnosis and MS type (i.e. benign, primary progressive and relapsing-remitting) was developed. Treatment and time (and their interaction) were specified as fixed effects, centre (three levels) and participant (nested in centre) as random effects in order to account for homogeneity within centre and within participant correlation over time. Initially a model containing the main effects of the treatment, time and a treatment-by-time interaction was specified in order to test whether there is evidence that the treatment effects varies over time. If the interaction was deemed unnecessary (using a likelihood ratio test) the model was refitted excluding the interaction term, so the treatment effect was then constant over time. All analyses were carried out using all available measurements. All models were fitted in R 3.2.0 using the lme4 and lmerTest packages. Model diagnostics involved suitable plots of the residuals.

Given increased calls across the literature to move beyond null-hypothesis significance testing in favour of effect sizes and confidence intervals [39] we also quantified and compared the magnitude of change in secondary outcomes using Hedges’ *g* effect sizes and associated 95% confidence intervals (95%CI) using Cohen’s conventions for effect sizes (0.2 small, 0.5 moderate, 0.8 large). For each outcome measure, the mean baseline to post intervention and 3 and 6 month change for the control condition was subtracted from the mean baseline to post intervention and 3 and 6 month change for the intervention condition and divided by the pooled baseline standard deviation [40]. Effect sizes were calculated such that greater improvements in outcomes in the intervention group compared to the control group resulted in positive effect sizes.

An exploratory paired t-test between baseline post intervention and 3- and 6- month follow-ups was also conducted. This provides a summary of the effects of the estimated treatment and control from the raw data. These “unadjusted” results do not account for the patient covariates and repeated measurements.

## Results

**Fig. 1:**
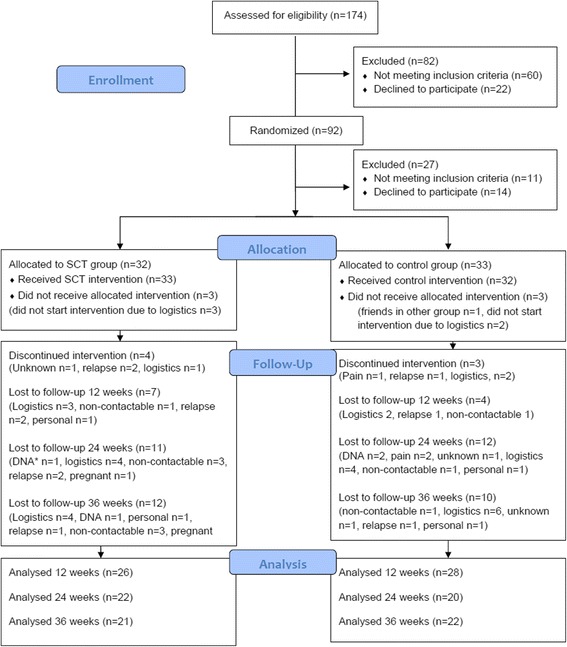
CONSORT Flow Diagram, DNA: did not attend

One hundred and seventy-four PwMS contacted the trial centre and were screened for inclusion over the phone between September 2013 and May 2014. Figure 1 illustrates the flow through the trial, including reasons for loss to follow-up and discontinuation of intervention. We randomised 92 individual participants and waited for 6 participants in a region to run a group before baseline assessment. While waiting for others to be randomised, 27 people became ineligible or declined to participate. One participant was not treated as randomised (two acquaintances had been randomised to the other group and the participant wanted to exercise with them). Sixty-five participants were assessed at baseline and commenced the intervention (intervention group *n* = 33, control group *n* = 32). Baseline demographics are presented in Table 1; the groups were similar at baseline. Feasibility, fidelity and adherence metrics are published elsewhere (Hayes et al. in press).

**Table 1:** Clinical baseline characteristics of those receiving exercise plus SCT (SCT) and exercise plus control education (CON)

	SCT (*n* = 33)	CON (*n* = 32)
MS type		
Benign	3	1
Primary progressive	1	0
Relapsing-remitting	27	27
Secondary progressive	0	1
Not reported	2	3
EDSS (mean, SD)	3.3 (0.7)	3.3 (0.7)
Years since diagnosis (mean, SD)	6.7 (5.7)	7.0 (6.1)
Centre (*n*)s		
Cork	10	9
Galway	8	10
Limerick	15	13
Age (years)	43.3 (9.9)	41.9 (9.3)
Gender (*n*)		
Male	4	6
Female	29	26

The raw data at each time point is presented in Table 2. Linear mixed effects models showed no statistically significant differences between the SCT and control groups for any secondary outcome at post intervention and at 3 and 6 month follow up assessment points (Table 2). Hedges’ g effect sizes and associated 95% CIs are also presented for each group in Table 3. At three month follow up, compared to the control group, the exercise and SCT education resulted in statistically significant moderate-to-large improvements in cognitive processing speed (SDMT: *g* = 0.75, 95% CI: 0.24, 1.25) and aerobic capacity (mCAFT: *g* = 0.51, 95% CI: 0.01, 1.00). Though not statistically significant, compared to control post intervention, the SCT group had small-to-moderate improvements in the perceived psychological impact of MS (*g* = 0.25), anxiety symptoms (*g* = 0.34), estimated energy expenditure (*g* = 0.39), exercise planning (*g* = 0.34), and social support (*g* = 0.40). Compared to control at three month follow-up, the SCT group had nonsignificant, small-to-moderate improvements in the perceived psychological impact of MS (*g* = 0.34), anxiety (*g* = 0.37) and depressive (*g* = 0.20) symptoms, lower extremity muscle strength (*g* = 0.41), estimated energy expenditure (*g* = 0.40), exercise planning (*g* = 0.31), exercise self-efficacy (*g* = 0.33), and social support (*g* = 0.49). At six month follow-up, compared to control the intervention showed nonsignificant small-to-moderate improvements in anxiety (*g* = 0.17) and depressive (*g* = 0.23) symptoms, cognitive processing speed (*g* = 0.15), lower extremity muscle strength (*g* = 0.49), aerobic capacity (*g* = 0.34), exercise planning (*g* = 0.17), exercise self-efficacy (*g* = 0.28), and social support (*g* = 0.45).

**Table 2:** Mean (SD) for secondary outcome measures at each time point

	Baseline mean (SD)		Post Intervention mean (SD)		Three month follow up mean (SD)		Six month follow up mean (SD)	
Variables	EXE + SCT	Control	EXE + SCT	Control	EXE + SCT	Control	EXE + SCT	Control
Modified Fatigue Impact Scale	43.6 (17.7)	46.1 (14.3)	37.2 (14.5)	37.7 (12.9)	35.7 (15.5)	37.6 (13.4)	37.9 (15.3)	36.4 (14.3)
Multiple Sclerosis Impact Scale29 - physical	29.6 (21.8)	30.0 (18.9)	25.6 (17.6)	27.2 (15.0)	24.3 (18.5)	24.4 (13.5)	25.8 (18.7)	25.8 (17.4)
Multiple Sclerosis Impact Scale29 psychological	39.8 (23.9)	36.1 (17.8)	29.4 (16.7)	30.9 (20.2)	29.7 (25.2)	33.1 (22.3)	35.3 (22.9)	29.2 (18.9)
Hospital Anxiety Depression Scale-Anxiety	8.2 (4.4)	7.3 (3.7)	6.6 (4.4)	7.1 (3.7)	6.2 (4.3)	6.8 (3.4)	6.4 (3.7)	6.2 (3.5)
Hospital Anxiety Depression Scale-Depression	5.9 (3.3)	5.5 (3.6)	4.9 (3.4)	4.9 (4.1)	4.1 (3.7)	4.4 (4.5)	4.8 (3.2)	5.2 (3.6)
Symbol Digit Modality Test	46.0 (10.2)	51.2 (14.2)	49.8 (9.1)	55.6 (12.6)	52.4 (7.5)	48.4 (14.1)	49.2 (9.5)	52.6 (15.8)
5xSit To Stand	11.5 (2.7)	10.8 (2.6)	9.8 (2.2)	9.4 (1.9)	9.9 (1.9)	10.3 (2.3)	9.3 (2.4)	9.9 (2.4)
Modified Canadian Aerobic Fitness Test	295.7 (54.6)	313.6 (59.0)	309.1 (53.8)	331.3 (51.6)	301.6 (50.2)	292.5 (53.5)	308.0 (51.5)	307.7 (51.9)
Godin Leisure Time Exercise Questionnaire – Health Index	3.0 (6.2)	2.8 (9.1)	13.8 (11.7)	16.1 (21.1)	15.2 13.9)	16.0 (16.7)	15.7 (14.8)	15.0 20.9)
Mean steps per day	6098.4 (2362.7)	7222.6 (2694.5)	6107.7 (3304.9)	7582.1 (2372.3)	6429.0 (2447.6)	7396.5 (2611.9)	6558.3 (3059.4)	7450.3 (3099.5)
Mean Energy Expenditure per day	1908.9 (299.8)	2031.6 (382.5)	1908.6 (372.4)	1897.4 (426.5)	1866.2 (407.7)	1851.5 (368.4)	1822.5 (348.16)	1899.7 (355.4)
Exercise Benefits Questionnaire	88.9 (9.1)	88.2 (8.4)	90.4 (12.1)	90.6 (11.2)	89.8 (12.6)	90.1 (11.7)	90.6 (12.5)	91.6 (11.8)
Exercise Barriers Questionnaire	29.8 (5.8)	29.8 (4.0)	28.1 (4.1)	28.5 (4.1)	28.7 (6.6)	27.5 (4.2)	28.7 (5.8)	28.0 (4.7)
Multidimensional Outcomes Expectations for Exercise Scale total	60.9 (6.9)	60.2 (5.9)	60.5 (7.4)	61.0 (7.3)	61.3 (7.0)	60.4 (6.0)	61.2 (7.9)	60.4 (6.9)
Exercise Goal setting Questionnaire	17.2 (7.9)	13.8 (6.6)	24.1 (10.2)	20.2 (7.7)	23.0 (10.5)	21.1 (7.8)	21.4 (10.1)	20.0 (9.9)
Exercise Planning Questionnaire	19.6 (6.3)	19.7 (5.3)	25.6 (6.7)	23.7 (7.4)	26.7(8.1)	25.0 (7.3)	24.9 (8.2)	24.0 (7.5)
Exercise Self-efficacy	66.3 (21.9)	69.2 (25.9)	66.0 (26.3)	70.5 (27.5)	69.0 (19.3)	63.9 (27.2)	68.7 (30.0)	64.9 (23.7)
Social Provisions Scale total	74.9 (9.7)	78.1 (9.1)	77.9 (8.3)	77.3 (9.0)	77.9 (10.2)	76.5 (10.6)	78.7 (9.6)	77.7 (10.1)

**Table 3:** Estimated treatment effects for secondary outcomes at each time point from linear mixed effects model and effect sizes

		Estimate of difference between SCT and Control	Standard error	95% CI	*p*-value	Hedges’ *g* (95% CI)
Modified Fatigue Impact Scale	Post Intervention Three month follow up Six month follow up	1.05 −0.11 4.39	3.32 3.49 3.41	(−5.56, 7.65) (−7.05, 6.82) (−2.38, 11.17)	0.75 0.97 0.20	−0.12 (−0.61, 0.36) −0.04 (−0.52, 0.45) −0.25 (−0.74, 0.24)
Multiple Sclerosis Impact Scale29 - physical	Post Intervention Three month follow up Six month follow up	−2.58 −2.34 −0.54	3.26 3.45 3.35	(−9.06, 3.89) (−9.19, 4.51) (−7.19, 6.10)	0.43 0.50 0.87	0.06 (−0.43, 0.55) −0.01 (−0.50, 0.47) −0.02 (−0.51, 0.47)
Multiple Sclerosis Impact Scale29 - psychological	Post Intervention Three month follow up Six month follow up	−3.01 −5.26 3.90	4.39 4.71 4.54	(−11.71, 5.70) (−14.59, 4.06) (−5.09, 12.90)	0.50 0.27 0.39	0.25 (−0.24, 0.73) 0.34 (−0.15, 0.83) −0.11 (−0.60, 0.37)
Hospital Anxiety Depression Scale-Anxiety	Post Intervention Three month follow up Six month follow up	−0.92 −0.93 0.01	0.91 0.95 0.93	(−2.72, 0.88) (−2.81, 0.92) (−1.83, 1.86)	0.31 0.33 0.99	0.34 (−0.15, 0.83) 0.37 (−0.12, 0.86) 0.17 (−0.32, 0.66)
Hospital Anxiety Depression Scale-Depression	Post Intervention Three month follow up Six month follow up	0.45 0.43 0.10	0.77 0.81 0.79	(−1.08, 1.97) (−1.18, 2.03) (−1.46, 1.66)	0.56 0.60 0.90	0.12 (−0.37, 0.60) 0.20 (−0.28, 0.69) 0.23 (−0.26, 0.72)
Symbol Digit Modality Test	Post Intervention Three month follow up Six month follow up	−3.03 4.93 −0.55	2.34 2.49 2.51	(−7.70, 1.64) (−0.02, 9.87) (−5.54, 4.44)	0.20 0.05 0.83	0.06 (−0.42, 0.55) **0.75 (0.24, 1.25)** 0.15 (−0.34, 0.63)
5xSit To Stand	Post Intervention Three month follow up Six month follow up	0.06 −0.09 −0.62	0.45 0.48 0.48	(−0.84, 0.97) (−1.10, 0.86) (−1.60, 0.33)	0.89 0.85 0.20	0.11 (−0.37, 0.60) 0.41 (−0.08, 0.91) 0.49 (−0.003, 0.98)
Modified Canadian Aerobic Fitness Test	Post Intervention Three month follow up Six month follow up	−7.02 16.12 10.53	9.75 10.63 10.90	(−26.45, 12.41) (−5.03, 37.26) (−11.13, 32.19)	0.47 0.13 0.34	−0.08 (−0.57, 0.40) **0.51 (0.01, 1.00)** 0.34 (−0.15, 0.83)
Godin Leisure Time Exercise Questionnaire – Health Index	Post Intervention Three month follow up Six month follow up	−2.24 0.42 0.11	4.55 4.75 4.64	(−11.30, 6.82) (−9.02, 9.87) (−9.12, 9.34)	0.62 0.93 0.98	−0.32 (−0.81, 0.17) −0.13 (−0.62, 0.36) 0.06 (−0.42, 0.55)
Mean steps per day	Post Intervention Three month follow up Six month follow up	−163.35 −439.08 448.87	696.12 703.49 737.27	(−1552.20, 1225.50) (−1842.34, 964.19) (−1020.74, 1918.48)	0.81 0.53 0.54	−0.14 (−0.63, 0.35) 0.06 (−0.42, 0.55) 0.08 (−0.57, 0.40)
Mean Energy Expenditure per day	Post Intervention Three month follow up Six month follow up	114.42 53.61 63.47	95.05 98.07 103.30	(−74.65, 303.48) (−141.39, 248.61) (−141.84, 268.78)	0.23 0.59 0.54	0.39 (−0.10, 0.88) 0.40 (−0.09, 0.89) 0.12 (−0.36, 0.61)
Exercise Benefits Questionnaire	Post Intervention Three month follow up Six month follow up	0.311 1.01 0.11	3.01 3.09 3.05	(−5.34, 6.23) (−5.16, 7.19) (−5.98, 6.20)	0.84 0.74 0.97	−0.10 (−0.59, 0.38) −0.11 (−0.60, 0.37) −0.19 (−0.68, 0.29)
Exercise Barriers Questionnaire	Post Intervention Three month follow up Six month follow up	−0.24 0.73 0.67	1.07 1.12 1.09	(−2.37, 1.88) (−1.50, 2.96) (−1.50, 2.85)	0.82 0.52 0.54	0.08 (−0.41, 0.57) −0.24 (−0.73, 0.25) −0.14 (−0.63, 0.35)
Multidimensional Outcomes Expectations for Exercise Scale total	Post Intervention Three month follow up Six month follow up	−0.42 1.31 1.13	1.56 1.63 1.59	(−3.52, 2.68) (−1.93, 4.55) (−2.03, 4.30)	0.79 0.42 0.48	−0.18 (−0.67, 0.30) 0.03 (−0.46, 0.52) 0.02 (−0.47, 0.50)
Exercise Goal setting Questionnaire	Post Intervention Three month follow up Six month follow up	2.03 −0.50 −0.26	2.15 2.30 2.21	(−2.24, 6.30) (−5.05, 4.06) (−4.65, 4.13)	0.35 0.83 0.91	0.07 (−0.42, 0.56) −0.21 (−0.69, 0.28) −0.27 (−0.76, 0.21)
Exercise Planning Questionnaire	Post Intervention Three month follow up Six month follow up	2.08 1.86 0.77	1.74 1.84 1.79	(−1.38, 5.34) (−1.78, 5.51) (−2.77, 4.32)	0.24 0.31 0.67	0.34 (−0.15, 0.83) 0.31 (−0.18, 0.80) 0.17 (−0.32, 0.66)
Exercise Self-efficacy	Post Intervention Three month follow up Six month follow up	−3.89 3.01 1.66	7.17 7.48 7.31	(−18.15, 10.38) (−11.86, 17.88) (−12.88, 16.20)	0.59 0.69 0.82	−0.07 (−0.55, 0.42) 0.33 (−0.16, 0.82) 0.28 (−0.21, 0.77)
Social Provisions Scale total	Post Intervention Three month follow up Six month follow up	1.69 2.79 1.15	1.82 1.91 1.86	(−1.94, 5.32) (−0.10, 6.58) (−2.55, 4.85)	0.36 0.15 0.54	0.40 (−0.09, 0.90) 0.49 (−0.005, 0.98) 0.45 (−0.05, 0.94)

**Bold text** indicates moderate effect (Heges G > 0.5)

Within-group outcome changes, including the unadjusted, unstandardized mean changes from baseline, associated 95%CIs, and paired t-test results for both groups, are presented in Table 4. Both groups demonstrated significant improvements from baseline following the 10-week intervention in the perceived impact of fatigue (MFIS), lower extremity muscle strength (5xSTS), self-reported PA (Godin Health Index), exercise goal setting, and exercise planning that are maintained above baseline at three and six month follow-up. Only the SCT group had significant improvements in perceived physical impact of MS (MSIS-29 physical), anxiety (HADS-A) and depressive (HADS-D) symptoms, and cognitive processing speed (SDMT) at three and six month follow-up. There was no significant change in objectively-measured PA using the outputs of steps and energy expenditure, and no significant change in exercise self-efficacy in either group across time points.

**Table 4:** Unadjusted comparisons of change in secondary outcome measures in each group at each time point

	Mean change Baseline to Post Intervention (95% CI) *p*-value		Mean change Baseline to three month follow up (95% CI) *p*-value		Mean change baseline to six month follow up (95% CI) *p*-value	
Variables	Intervention	Control	Intervention	Control	Intervention	Control
Modified Fatigue Impact Scale	**−4.99** **(−9.78, −0.21)** ***p*** **= 0.04**	**−7.68** **(−12.13, −3.23)** ***p*** **= 0.01**	**−8.09** **(−13.97, −2.20)** ***p*** **< 0.01**	**−7.41** **(−12.77, −2.04)** ***p*** **< 0.01**	**−5.22** **(−10.99, 0.55)** ***p*** **= 0.07**	**−10.35** **(−16.43, −4.27)** ***p*** **< 0.01**
Multiple Sclerosis Impact Scale29 - physical	−2.06 (−6.36, 2.23) *p* = 0.33	−0.13 (−5.63, 5.37) *p* = 0.96	**−4.45** **(−8.68, −0.22)** ***p*** **= 0.04**	−1.42 (−7.69, 4.84) *p* = 0.64	**−4.12** **(−8.25, 0.01)** ***p*** **= 0.05**	−3.61 (−9.83, 2.62) *p* = 0.24
Multiple Sclerosis Impact Scale29 psychological	**−7.54** **(−13.28, −1.81)** ***p*** **= 0.01**	−4.22 (−11.19, 2.76) *p* = 0.22	**−8.37** **(−15.85, −0.89)** ***p*** **= 0.03**	−2.02 (−8.11, 4.07) *p* = 0.50	−4.94 (−12.84, 2.98) *p* = 0.21	**−7.60** **(−13.71, −1.49)** ***p*** **= 0.02**
Hospital Anxiety Depression Scale-Anxiety	−1.60 (−2.50, 0.30) *p* = 0.12	−0.35 (−1.61, 0.90) *p* = 0.57	**−1.76** **(−3.20, −0.31)** ***p*** **= 0.02**	−0.51 (−1.94, 0.92) *p* = 0.47	**−1.99** **(−3.28, −0.71)** ***p*** **< 0.01**	−1.26 (−2.67, 0.15) *p* = 0.08
Hospital Anxiety Depression Scale-Depression	−0.76 (−1.54, 0.33) *p* = 0.20	−0.61 (−1.54, 0.33) *p* = 0.19	**−1.51** **(−2.89, −0.13)** ***p*** **= 0.03**	−1.02 (−2.50, 0.47) *p* = 0.17	**−1.02** **(−2.05, 0.01)** ***p*** **= 0.05**	−0.37 (−1.35, 0.61) *p* = 0.44
Symbol Digit Modality Test	1.65 (−1.68, 4.99) *p* = 0.32	3.43 (−1.01, 7.87) *p* = 0.12	**5.04** **(2.51, 7.57)** ***p*** **< 0.01**	0.24 (−3.71, 4.18) *p* = 0.90	**3.05** **(0.81, 5.28)** ***p*** **< 0.01**	2.56 (0.07, 5.06) *p* = 0.04
5xSit To Stand	**−1.51** **(−2.41, −0.60)** ***p*** **< 0.01**	**−1.55** **(−2.30, −0.79)** ***p*** **< 0.01**	**−2.13** **(−3.00, −1.26)** ***p*** **< 0.01**	**−1.49** **(−2.32, −0.65)** ***p*** **< 0.01**	**−2.29** **(−3.20, −1.39)** ***p*** **< 0.01**	**−1.19** **(−1.97, −0.40)** ***p*** **< 0.01**
Modified Canadian Aerobic Fitness Test	8.56 (−6.86, 23.98) *p* = 0.26	10.54 (−6.29, 27.37) *p* = 0.21	3.40 (−14.07, 20.87) *p* = 0.69	−11.97 (−33.02, 9.08) *p* = 0.25	2.87 (−7.18, 13.54) *p* = 0.58	−11.54 (−27.67, 4.59) *p* = 0.15
Godin Leisure Time Exercise Questionnaire – Health Index	**9.85** **(5.46, 14.23)** ***p*** **< 0.01**	**12.92** **(4.69, 20.89)** ***p*** **< 0.01**	**10.87** **(4.20, 17.53)** ***p*** **< 0.01**	**12.35** **(5.63, 19.07)** ***p*** **< 0.01**	**10.65** **(4.18, 17.12)** ***p*** **< 0.01**	**11.67** **(5.67, 17.67)** ***p*** **< 0.01**
Mean steps per day	118.67 (−1002.54, 1239.87) *p* = 0.83	30.38 (−1116.58, 1177.34) *p* = 0.96	−5.88 (−940.73, 928.97) *p* = 0.99	96.85 (−621.18, 814.88) *p* = 0.78	756.92 (−440.88, 1954.73) *p* = 0.19	86.28 (−849.85, 1022.42) *p* = 0.85
Mean Energy Expenditure per day	46.22 (−119.73, 212.17) *p* = 0.56	−72.90 (−224.14, 78.33) *p* = 0.33	−58.18 (−162.68, 46.33) *p* = 0.25	**−182.5** **(−335.03, −29.97)** ***p*** **= 0.02**	6.61 (−140.78, 154.01) *p* = 0.92	−66.76 (−212.64, 79.12) *p* = 0.35
Exercise Benefits Questionnaire	2.64 (−0.53, 5.80) *p* = 0.09	2.08 (−2.31, 6.48) *p* = 0.34	2.18 (−2.41, 6.78) *p* = 0.33	1.83 (−3.21, 6.87) *p* = 0.46	3.38 (−0.59, 7.35) *p* = 0.09	2.39 (−2.33, 7.12) *p* = 0.31
Exercise Barriers Questionnaire	**−1.51** **(−2.86, −0.17)** ***p*** **= 0.03**	−1.09 (−2.41, 0.24) *p* = 0.10	−1.27 (−3.35, 0.82) *p* = 0.22	−1.51 (−3.40, 0.37) *p* = 0.11	−1.57 (−3.22, 0.07) *p* = 0.06	−2.11 (−4.24, 0.02) *p* = 0.05
Multidimensional Outcomes Expectations for Exercise Scale total	1.17 (−0.87, 3.21) *p* = 0.25	0.86 (−1.46, 3.19) *p* = 0.45	1.84 (−0.24, 3.93) *p* = 0.08	−0.21 (−2.87, 2.44) *p* = 0.87	1.84 (−0.35, 4.04) *p* = 0.10	−0.70 (−3.09, 1.68) *p* = 0.55
Exercise Goal setting Questionnaire	**7.30** **(4.19, 10.4)** ***p*** **< 0.01**	**5.96** **(2.92, 9.01)** ***p*** **< 0.01**	**5.44** **(2.14, 8.74)** ***p*** **< 0.01**	**6.38** **(2.76, 10.0)** ***p*** **< 0.01**	**4.88** **(2.01, 7.76)** ***p*** **< 0.01**	**5.51** **(1.89, 9.13)** ***p*** **< 0.01**
Exercise Planning Questionnaire	**5.88** **(3.37. 8.39)** ***p*** **< 0.01**	**3.76** **(1.27, 6.25)** ***p*** **< 0.01**	**6.42** **(3.89, 8.96)** ***p*** **< 0.01**	**4.68** **(1.76, 7.61)** ***p*** **< 0.01**	**4.86** **(2.63, 7.09)** ***p*** **< 0.01**	**4.20** **(1.55, 6.86)** ***p*** **< 0.01**
Exercise Self-efficacy	−0.06 (−12.20, 12.07) *p* = 0.99	0.52 (−11.75, 12.79) *p* = 0.93	1.32 (−10.62, 13.26) *p* = 0.82	−9.42 (−24.03, 5.19) *p* = 0.19	4.24 (−7.78, 16.25) *p* = 0.47	−5.62 (−15.65, 4.42) *p* = 0.26
Social Provisions Scale total	1.66 (−1.13, 4.44) *p* = 0.23	−0.20 (−2.65, 2.25) *p* = 0.86	2.29 (−0.08, 4.65) *p* = 0.06	−0.66 (−4.61, 3.29) *p* = 0.73	**3.13** **(0.35, 5.90)** ***p*** **= 0.03**	0.77 (−2.18, 3.72) *p* = 0.60

**Bold text** indicates statistical significance *p* < 0.05

## Discussion

This paper presents the secondary outcome results from an intervention designed to enable inactive people with MS to reach the minimum recommendation of the MS Exercise Guidelines and further compared the effect of a structured SCT-based education to an attention-control education intervention. Null hypothesis testing demonstrated no statistically significant between-group differences for any secondary outcome over time. However, examination of the magnitude of change quantified by Hedges’ *g* effect sizes illustrated potentially important differences between exercise plus SCT compared to the attention control condition, including significant moderate-to-large improvements in cognitive processing speed and aerobic capacity at three month follow-up. Additionally, though not statistically significant, compared to the attention-control condition, exercise plus SCT resulted in small-to-moderate improvements of ¼ to ½ standard deviation in anxiety and depressive symptoms, the perceived psychological impact of MS, cognitive processing speed, aerobic capacity, estimated energy expenditure, exercise planning, and social support, and the magnitude of many of these improvements persisted at 24- and 36-week follow-up. The magnitude of improvements in these outcomes is consistent with previous reports of the positive effects of exercise training on symptoms among PwMS, including fatigue [1], anxiety [41], depression [42, 43], quality of life [7, 44], and mobility [5], and highlights the potential additive benefit of combined SCT-based education and exercise training.

The finding that both groups improved in strength and physical activity is not surprising given the content of the intervention. Participants completed twice weekly resistance exercise and moderate intensity walking exercise. The changes in lower extremity muscle strength 6 months post intervention, measured with the 5xSTS test, are in line broadly with previously reported changes using that measure [45], providing support for the fidelity of the current intervention to enhance strength. The Health Index score of the GLTEQ also increased in both groups, confirming the exercise log data which indicated that SCT and the CON group groups completed an average of 33.2 of 44 available sessions (75.5%) and 32.0 sessions (72.6%), respectively (Hayes et al. in press). Of note, the objective measure of PA, mean steps/day and mean energy expenditure/day did not change in line with the positive effects on walking mobility and the increase in PA reported in the exercise logs and the GLTEQ Health Index. This may be due to reduced non-exercise physical activity, such that participants reduced leisure, transport and occupational PA in order to engage in exercise training, thereby maintaining, or even decreasing, their overall PA levels. There has been some support in the literature for initial decreases in non-exercise physical activity when beginning an exercise training intervention, though the available evidence suggests that decreased activity dissipates with continued training [46]. It is also possible that the arm worn accelerometer did not capture the changes in PA or that an alternative output, such as increased mild/moderate PA specifically or a reduction in sedentary behaviour, may capture the changes due to the intervention.

Importantly, the results of within-group changes confirmed the positive effect of exercise on fatigue for people with MS [1, 2]. The included sample of PwMS started with scores on the MFIS greater than 38 [47], indicative of clinically meaningful fatigue, and both groups improved, reporting scores below 38 at three and 6 month follow up. To the authors’ knowledge, this is the first study to confirm that exercising at the minimum recommendations of the Canadian MS Exercise guideline [9] has a positive effect on fatigue for inactive people with MS with mild-moderate disability.

Both groups also improved in exercise goal setting and planning and these improvements were maintained at both follow-up assessments. This was expected in the SCT group because the structured education intervention specifically addressed these and other SCT domains. The improvement in the control group was unexpected as they engaged in didactic education on topics unrelated to exercise. On reflection, in our efforts to document adherence and fidelity to the intervention, we inadvertently provided the control group with several physical activity behaviour change techniques (BCT’s) [12]. This involved exercising in a group setting, advice on the guideline amount and its benefits, recording exercise in a log, seeing personal improvements and monitoring step count on a pedometer. Participants further reported in our qualitative data that knowing they were going to be assessed at 3- and 6-months further served as a motivator to keep exercising. These, somewhat “simple”, BCT’s warrant consideration for inclusion in interventions that aim to enable long term exercise behaviour and its benefits though we note that there was a greater improvement in the SCT group for the primary outcome of walking endurance seen in a per protocol analysis (Hayes et al. in press). Adding a booster session with both assessment and intervention in the follow up periods may further maintain exercise behaviour and its associated outcomes and is in line with a recent systematic review [48] and reports from participants in this study.

Interestingly exercise self efficacy did not change neither did outcome expectancies or exercise benefits. Notably, compared to the control condition, exercise plus SCT resulted in small, nonsignificant improvements in self-efficacy and exercise plus SCT also resulted in near half-standard deviation improvements in social support, measured with the Social Provisions Scale, at all time points. Though the objective of the current study was not to examine plausible mediators of the effects of exercise plus SCT on outcomes, given previous evidence of the potential intermediary role of social support in the effects of physical activity and exercise among PwMS [49], the ability of exercise to concurrently improve social support and symptoms may be particularly important.

Interestingly only the SCT group demonstrated improvements in physical impact of MS, anxiety, depression and cognition. Compared to the attention-control condition, the exercise plus SCT group showed small-to-moderate improvements in anxiety and depressive symptoms, the perceived psychological impact of MS, and cognitive processing speed. These findings warrant further focused examination but may be due in part to the greater change in walking mobility seen in the exercise plus SCT group (Hayes et al. in press). Both groups improved significantly in six minute walk test (6MWT) distance after treatment and at 3- and 6-month follow-up, and using intention-to-treat analysis the SCT group demonstrated 22.70 m, 11.80 m and 27.42 m greater improvements in this measure. Data suggest that a 21 m change is clinically meaningful to participants, supporting the hypothesis that the SCT group had more meaningful changes in 6MWT distance at 3- and 6-month follow-up that may have resulted in reduced physical impact of MS. More of the SCT group reached the guideline and they reported completing more sessions. This increased “total exercise dose” and the resulting change in depression is supported by our recent systematic review and meta-regression analysis [42], which found increased frequency of exercise was associated with greater reductions of depression. There is limited specific information on the dose/response relationship of exercise for MS and this warrants consideration in trials designed specifically to address this question.

### Strengths and limitations

The main strength of this study is that we purposely recruited inactive people with MS and engaged them in a 10-week exercise and education programme with the aim of enabling long-term physical activity engagement and its associated benefits. A weakness is that we did not power the study for these secondary outcomes; nonetheless, the preliminary findings presented in the current paper, particularly the magnitude of improvements in fatigue, anxiety and depressive symptoms, the perceived impact of MS, strength and aerobic capacity, and cognitive processing speed, will inform future trials and targeted analyses of these important factors. A further strength is that we measured a broad range of MS symptoms, and reported the effect of the resistance and aerobic exercise programme on strength, fitness, and subjectively and objectively measured PA. A limitation is that the measures of objective PA and fitness did not change and a more direct measure of fitness, such as cycle ergometry to determine VO^2^ max, is recommended [50]. Participants reported some dissatisfaction with the SWA arm band; therefore, alternative tools for objectively measuring PA among PwMS are warranted. A further strength is that we used exercise logs to capture intensity of aerobic exercise using steps from a pedometer, but a limitation is that we did not record heart rate and this is recommended in future trials.

## Conclusion

This paper presents data to suggest that enabling inactive people with mild-moderate disability due to MS to exercise at the minimum suggested by the exercise guideline results in a range of benefits. Improvements in fatigue, strength, goal setting and planning were seen in both the structured SCT and attention control groups and were maintained at 3 and 6 month follow up. The similar responses in both groups for these secondary outcomes can be explained as they both had the same exercise intervention and by the inadvertent inclusion of several behaviour change techniques for the control group through our adherence logging and trial structure.

The results of this pilot trial for the secondary outcome measures suggest that the SCT group had greater improvements in cognitive processing and aerobic capacity at 3 month follow up. This paper further presents preliminary evidence for improvements in physical impact of MS, anxiety, depression and cognition in the exercise plus SCT group alone. This may in part be due to the greater improvements in walking mobility reported elsewhere or due to the content of the education element and therefore further testing of the intervention model is warranted. These findings, in combination with the effects for the primary outcome measure, warrant progression to a definitive RCT and suggest the importance of studies directly investigating the dose-response relationship with focal outcomes.

## Abbreviations

5xSTS: 5 times sit to stand test
6MWT: Six minute walk test
BCT: Behaviour change technique
EDSS: Expanded disability status scale
EXSE: Exercise self-efficacy scale
GLTEQ: Godin leisure-time exercise questionnaire
HADS: Hospital anxiety and depression scale
mCAFT: Modified Canadian aerobic fitness test
MFIS: Modified fatigue impact scale
MOEES: Multidimensional outcomes expectations for exercise scale
MS: Multiple sclerosis
MSIS-29: Multiple sclerosis impact scale 29
PDDS: Patient determined disease steps
PwMS: People with multiple sclerosis
QOL: Quality of life
RCT: Randomised controlled trial
SCT: Social cognitive theory
SDMT: Symbol digit modalities test
SPS: Social provisions scale
SWA: SenseWear Armband
